# Fibroblast growth factor 21 deletion aggravates diabetes-induced pathogenic changes in the aorta in type 1 diabetic mice

**DOI:** 10.1186/s12933-015-0241-0

**Published:** 2015-06-11

**Authors:** Xiaoqing Yan, Jun Chen, Chi Zhang, Jun Zeng, Shanshan Zhou, Zhiguo Zhang, Xuemian Lu, Jing Chen, Wenke Feng, Xiaokun Li, Yi Tan

**Affiliations:** Chinese-American Research Institute for Diabetic Complications at the Wenzhou Medical University, Wenzhou, China; Chinese-American Research Institute for Pediatrics of the First Affiliated Hospital at the Wenzhou Medical University, Wenzhou, China; Department of Endocrinology, the Third Hospital Affiliate to Wenzhou Medical University, Ruian, China; Kosair Children’s Hospital Research Institute, the Department of Pediatrics of the University of Louisville, School of Medicine, Louisville, USA; School of Nursing, Wenzhou Medical University, Wenzhou, China; Departments of Cardiovascular Disorders and Geriatrics of the First Hospital of Jilin University, Changchun, China; Department of Pharmacology and Toxicology of the University of Louisville School of Medicine, Louisville, USA

**Keywords:** Fibroblast growth factor 21, Vascular damage, Diabetes, Oxidative stress

## Abstract

Fibroblast growth factor 21 (FGF21) is an important regulator in glucose and lipid metabolism, and has been considered as a potential therapy for diabetes. The effect of FGF21 on the development and progression of diabetes-induced pathogenic changes in the aorta has not currently been addressed. To characterize these effects, type 1 diabetes was induced in both FGF21 knockout (FGF21KO) and C57BL/6 J wild type (WT) mice via multiple-dose streptozotocin injection. FGF21KO diabetic mice showed both earlier and more severe aortic remodeling indicated by aortic thickening, collagen accumulation and fibrotic mediator connective tissue growth factor expression. This was accompanied by significant aortic cell apoptosis than in WT diabetic mice. Further investigation found that FGF21 deletion exacerbated aortic inflammation and oxidative stress reflected by elevated expression of tumor necrosis factor α and transforming growth factor β, and the accumulation of 3-nitrotyrocine and 4-Hydroxynonenal. FGF21 administration can reverse the pathologic changes in FGF21KO diabetic mice. These findings demonstrate that FGF21 deletion aggravates aortic remodeling and cell death probably via exacerbation of aortic inflammation and oxidative stress. This marks FGF21 as a potential therapy for the treatment of aortic damage due to diabetes.

## Introduction

Diabetic vascular complications, including macroangiopathy, microangiopathy and peripheral vascular complications, are the most common diabetic complications in both type 1 [1] and type 2 [2] diabetes mellitus and make major contributions to diabetic mortality and morbidity [2]. Diabetic microvascular disease is a leading cause of blindness, renal failure and nerve damage. Furthermore, diabetic macroangiopathy and peripheral vascular complications lead to increased risk of myocardial infarction, stroke and limb amputation [3]. About 80 % of all diabetic patients die from cardiovascular events. Of which, 75 % are due to coronary heart disease and the remaining 25 % are attributed to cerebrovascular, peripheral or other macrovascular disease [4].

Even though the exact mechanism for accelerated vascular disease in diabetes is not yet fully clear, existing research has defined numerous risk factors involved in diabetes, such as oxidative stress [5, 6], dyslipidemia [4, 5], advanced glycation [7], decline in nitric oxide production, activation of the renin-angiotensin aldosterone system, and endothelial inflammation [4]. All contribute to the development of diabetic vascular complications.

Fibroblast growth factor 21 (FGF21), a newly-defined member of the FGF family [8], has been identified as a potent metabolic regulator with specific effects on glucose and lipid metabolism [9]. FGF21 can stimulate glucose uptake in adipocytes [10], and enhance glucose clearance by enhancing the browning of white adipose tissues [11]. In response to fasting, FGF21 can regulate lipolysis in adipocytes [12]. FGF21 also shows beneficial effects on lipid profiles as demonstrated by lower circulating lipids in both rodent [13] and primate [14] diabetic models following FGF21 administration. FGF21 treatment also enhanced expression and secretion of the downstream effector, adiponectin, in adipocytes, which in turn further improved fatty acid oxidation and lipid clearance in the liver and skeletal muscle [15]. Moreover, FGF21 has an insulin-sensitizing ability [15] and can ameliorate glucose tolerance [16] by reducing hepatic glucose production and stimulating glucose uptake in adipocytes.

Because of its ability to regulate glucose and lipid metabolism, FGF21 has shown therapeutic potential in treating diabetes [17]. FGF21 transgenic mice were lean and resistant to age-associated or diet-induced obesity and insulin resistance [13]. Both acute [18] and chronic [14] administration of FGF21 can ameliorate the metabolic state of diabetes. FGF21 treatment resulted in rapid decline of blood glucose levels and immediate improvement of glucose tolerance and insulin sensitivity in both *ob/ob* and diet-induced obese mice [18, 19] over the short term and ameliorated fasting hyperglycemia in both *ob/ob* mice [19] and diabetic monkeys [14] over the long term treatment. In addition, the level of serum FGF21 is reported to be positively associated with coronary artery disease [20] and higher risk of cardiovascular events in patients with type 2 diabetes [21], which might indicate a compensatory response. However, the direct effects of FGF21 on diabetic complications still remain largely unknown.

Almost all specific risk factors of diabetic vascular complications are directly related to hyperglycemia [1] and/or hyperlipidemia [2]. Ameliorating glucose and lipid metabolism is still a major preventive and assistive therapeutic strategy for diabetic vascular complications. Considering the anti-hyperglycemic and anti-hyperlipidemic effects of FGF21 on diabetes, and the fact that its preferred receptor, fibroblast growth factor receptor 1c (FGFR1c), and co-receptor, β-klotho, are highly-expressed in aorta [22], FGF21 is indicated to be involved in pathogenic changes in the aorta under diabetic conditions. Therefore, we investigated the role of FGF21 in the development and progression of pathogenic changes in the aorta in a streptozotocin (STZ)-induced type 1 diabetic model using FGF21 knockout (FGF21KO) mice.

## Materials and methods

### Ethic statement

This study was carried out in strict accordance with the recommendations in the Guide for the Care and Use of Laboratory Animals of the National Institutes of Health. The protocol was approved by the Animal Policy and Welfare Committee of Wenzhou Medical University and the Institutional Animal Care and Use Committee of the University of Louisville. All surgeries were performed under anesthesia induced by intraperitoneal injection of 1.2 % 2,2,2-Tribromoethanol (Avertin) at the dose of 300 mg/kg body weight and all efforts were made to minimize suffering.

### Animal model

The present study used male FGF21KO mice with C57 BL/6 J background (gifted by Dr. Steve Kliewer, University of Texas Southwestern Medical Center) [23] and wild type (WT) C57 BL/6 J mice purchased from Jackson Laboratory (Bar Harbor, Maine). The type 1 diabetes model was induced in 10 week-old male FGF21KO mice and age-matched WT mice by intraperitoneal injection of 6 consecutive doses of STZ (60 mg/kg body weight, Sigma, St. Louis, MO) in 10 mM sodium citrate buffer, pH 4.5. FGF21KO and WT mice control groups (Ctrl) received citrate buffer alone. Seven days after the last STZ injection, whole blood glucose obtained from the mouse tail vein was assayed using a SureStep complete blood glucose monitor (LifeScan, Milpitas, CA). Animals with blood glucose levels greater than 250 mg/dL were considered diabetic. At 1, 2 and 4 months following diabetes onset, mice were sacrificed and aorta tissue was collected.

In FGF21 treatment experiment, an acute type 1 diabetic model was induced in 10 week-old male FGF21KO mice and age-matched WT mice as described above. FGF21KO and WT mice control groups (Ctrl) received citrate buffer alone. FGF21KO diabetic mice in FGF21 treatment group received intraperitoneal injection of FGF21 (100 μg/kg body weight per day) for 2 months. Thereafter, mice were sacrificed and aorta tissue was collected.

### Aorta sample preparation and histopathological examination

Under anesthesia, thoracotomies were performed on mice and the descending thoracic aortas were carefully harvested and fixed in 10 % buffered formalin. Next, aorta tissues were cut into ring segments (2–3 mm in length), dehydrated in graded alcohol, cleared with xylene, and finally embedded in paraffin. Sections (5 μm thickness) were cut for pathological and immunohistochemical staining. Histological changes in the aorta were evaluated by hematoxylin and eosin (H&E) staining using Image Pro Plus 6.0 software for measuring the means of the tunica media width as the thickness of aortic tunica media.

### Sirius-red staining for collagen

Aortic fibrosis was evaluated by Sirius-red staining, as described previously [24]. Briefly, 5 μm tissue sections were stained with 0.1 % Sirius-red F3BA and 0.25 % Fast Green FCF and assessed for the proportion of collagen using a Nikon Eclipse E600 microscopy system.

### TUNEL staining

Terminal deoxynucleotidyl-transferase-mediated dUTP nick-end labeling (TUNEL) staining was performed on formalin-fixed, paraffin-embedded sections with Peroxidase *In Situ* Apoptosis Detection Kit (Millipore, Billerica, MA) according to the manufacturer’s instructions and nuclei were stained using methyl green (FD Neurotechnologies, Columbia, MD). Positively stained apoptotic cells were counted randomly in a minimum of five microscopic fields in each of the three slides per aorta under light microscopy. The percentage of TUNEL positive cells relative to 100 nuclei was presented.

### Immunohistochemical staining

Formalin-fixed, paraffin-embedded aorta sections were dewaxed using xylene and rehydrated by serial washes in graded alcohol and a final wash in dH_2_O for 15 min. After balanced with phosphate buffered saline (PBS), aorta sections were incubated in Target Retrieval buffer (DAKO, Carpinteria, CA) at 95 °C, and endogenous peroxidase was quenched by incubating in 3 % H_2_O_2_ at room temperature for 10 min. After washing with PBS 3 times, sections were blocked in 5 % bovine serum albumin (BSA) for 30 min, then incubated with primary antibody against mice tumor necrosis factor α (TNF-α), connective tissue growth factor (CTGF), transforming growth factor β (TGF-β), 3-nitrotyrocine (3-NT), 4-Hydroxynonenal (4-HNE), nuclear factor E2-related factor-2 (Nrf2) or phosphorylated endothelial nitric oxide synthase (p-eNOS, Ser 1177) overnight at 4 °C. Sections incubated with PBS were used as negative controls. After washing, sections were incubated with corresponding secondary antibodies at room temperature for 1 h. For the development of color, sections were treated with peroxidase substrate DAB kit (Vector Laboratories, Inc. Burlingame, CA) and counterstained with hematoxylin. Quantitative analysis was carried out using Image J software.

### Enzyme linked immunosorbent assay (ELISA)

Whole blood was collected in a lithium heparin tube (BD, Franklin Lakes, NJ) and centrifuged at 2000 rpm for 20 min. Plasma was used for interleukin- 6 (IL- 6) assay using a mouse IL-6 ELISA kit (Invitrogen, Frederick, MD) according to the manufacturer’s instructions.

### Statistical analysis

Data were collected from several animals (*n*=5 ~ 9) and presented as means ± SD. Image Pro Plus 6.0 software was used to measure pathological changes as described above. Comparisons were performed by one-way ANOVA for the different groups, followed by *post hoc* pairwise repetitive comparisons using Tukey’s test. Statistical analysis was done using Origin 7.5 Lab data analysis and graphing software. Statistical significance was considered as P <0.05.

## Results

### FGF21 deletion accelerated diabetes-induced aortic thickening

**Fig. 1:**
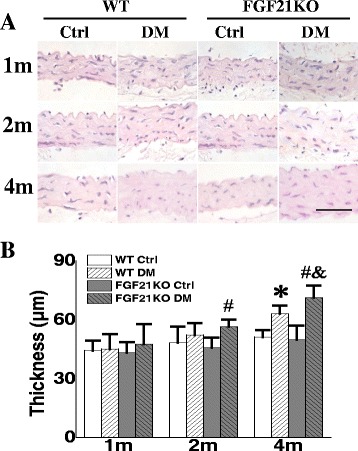
FGF21 deletion accelerated and aggravated diabetes-induced aortic thickening. At indicated time points after diabetes onset, histological change of aorta was evaluated by H&E staining (**a**) and aorta thickness was measured using Image Pro Plus 6.0 software (**b**). Data are presented as means ± SD, n ≥ 5 for each group. * *p* < 0.05 vs WT Ctrl group; # *p* < 0.05 vs FGF21KO Ctrl group; & *p* < 0.05 vs WT DM group. Bar = 50 μm. Ctrl: control; DM: diabetes mellitus; WT: wild type; FGF21KO: FGF21 knockout; m: month(s)

Thickening is one of the major pathologic changes in diabetic aorta [24]. At 1, 2 and 4 months after diabetes onset, aortic thickening was evaluated by H&E staining and thickness was measured using Image J software. Under non-diabetic conditions, FGF21KO mice did not show marked alterations in aortic wall thickness compared to the WT controls. Both WT and FGF21KO diabetic mice showed aortic wall thickness changes. However, WT diabetic mice only exhibited aortic wall thickening at the 4^th^ month after diabetes onset, while FGF21KO diabetic mice developed aortic wall thickening at the 2^nd^ month after diabetes onset with more severe aortic wall thickening than WT diabetic mice at the 4^th^ month after diabetes onset (Fig. 1a&b).

### FGF21 deletion aggravated diabetes-induced aortic fibrosis

**Fig. 2:**
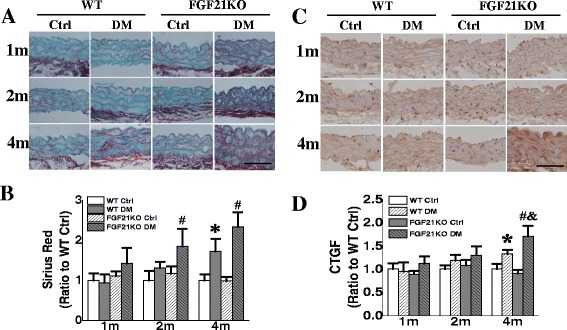
FGF21 deletion accelerated and aggravated diabetes-induced aortic fibrosis. At indicated time points after diabetes onset, aortic fibrosis was evaluated by Sirius Red staining of collagen accumulation (**a, b**) and immunohistochemical staining of CTGF expression (**c, d**). Data are presented as means ± SD, n ≥ 5 for each group. * *p* < 0.05 vs WT Ctrl group; # *p* < 0.05 vs FGF21KO Ctrl group; & *p* < 0.05 vs WT DM group. Bar = 50 μm. Abbreviations are the same as the Fig. 1

Fibrosis is another major pathologic change in diabetic macroangiopathy [24–26]. Sirius red staining demonstrated that FGF21 deletion did not increase collagen accumulation under non-diabetic conditions compared to the WT control (Fig. 2). WT diabetic mice did not show obvious collagen accumulation until the 4^th^ month after diabetes onset. But diabetes significantly accelerated and aggravated collagen accumulation in FGF21KO mice at 2 months after diabetes onset (Fig. 2a&b).

The aggravated fibrosis was also confirmed by immunohistochemical staining for the fibrotic mediator, CTGF. It was demonstrated that diabetes significantly up-regulated CTGF expression in both WT and FGF21KO diabetic mice at 4 months compared to their corresponding controls. This was significantly higher in FGF21KO diabetic mice than in WT diabetic mice (Fig. 2c&d). However, FGF21 deletion had no significant effect on CTGF expression under non-diabetic conditions compared to WT control mice at all 3 time points.

### FGF21 deletion exacerbated diabetes-induced aortic inflammation

**Fig. 3:**
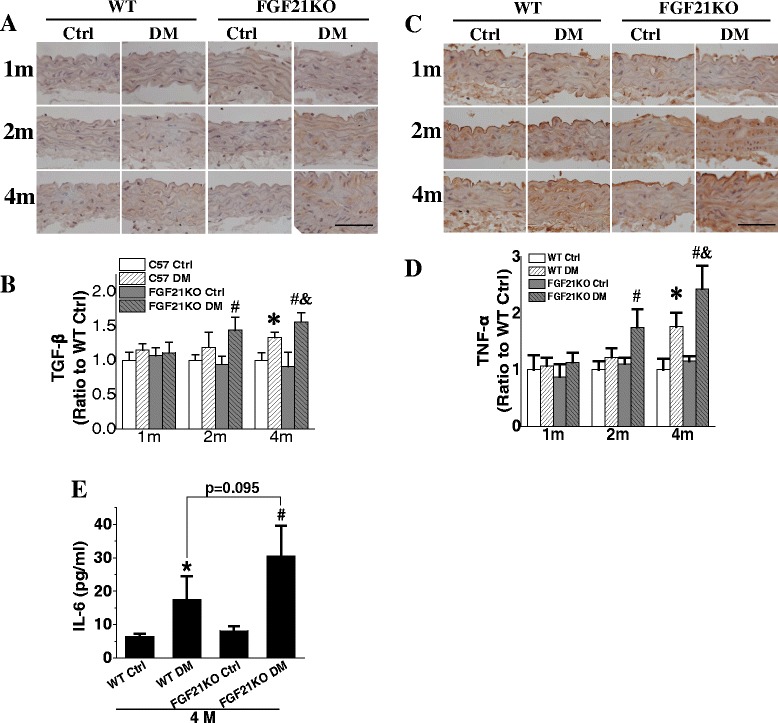
FGF21 deficiency aggravated diabetes-induced inflammation. At indicated time points after diabetes onset, aortic inflammation was evaluated by immunohistochemical staining of TGF-β expression (**a, b**) and TNF-α (**c, d**). Plasma IL-6 was detected by ELISA (**e**). Data are presented as means ± SD, n ≥ 5 for each group. * *p* < 0.05 vs WT Ctrl group; # *p* < 0.05 vs FGF21KO Ctrl group; & *p* < 0.05 vs WT DM group. Bar = 50 μm. Abbreviations are the same as the Fig. 1

Inflammation is an important cause of the pathologic changes in aorta under diabetic conditions [25]. Immunohistochemical staining showed a significant increase in inflammatory markers TGF-β and TNF-α expression in aortic tunica media of diabetic mice (Fig. 3). Both WT and FGF21KO diabetic mice had elevated TGF-β expression at the 4^th^ month compared to their corresponding control mice, and the aortic expression of FGF21KO in diabetic mice was significantly higher than that of WT diabetic mice (Fig. 3a&b). TNF-α expression was also elevated in WT diabetic mice at the 4^th^ month after diabetes onset compared to WT control mice. Furthermore, its expression was up-regulated at the 2^nd^ month after diabetes onset in FGF21KO mice and was much higher than that of WT diabetic mice at the 4^th^ month after diabetes onset (Fig. 3c&d). Under non-diabetic conditions, both TGF-β and TNF-α expression maintained their low levels and no differences between FGF21KO and WT mice were observed. In addition, FGF21 deletion dramatically up-regulated plasma IL-6 content under diabetic conditions (Fig. 3e), which indicated an aggravated systemic inflammation in FGF21KO diabetic mice.

### FGF21 deletion aggravated diabetes-induced aortic cell apoptosis

**Fig. 4:**
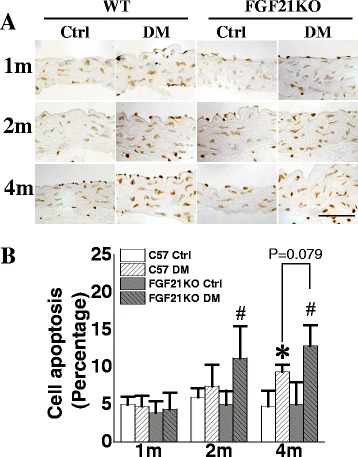
FGF21 deficiency accelerated diabetes-induced cell apoptosis. At indicated time points after diabetes onset, cell apoptosis was evaluated by TUNEL staining (**a, b**). Data are presented as means ± SD, n ≥ 5 for each group. * *p* < 0.05 vs WT Ctrl group; # *p* < 0.05 vs FGF21KO Ctrl group. Bar = 50 μm. Abbreviations are the same as the Fig. 1

Effect of FGF21 deletion on aortic cell apoptosis was evaluated by TUNEL staining. Obvious aortic cell apoptosis was observed in tunica intima and media in both WT and FGF21KO diabetic mice (Fig. 4). WT diabetic mice showed significant aortic cell apoptosis at the 4^th^ month after diabetes onset compared to the WT control mice. This phenomenon was observed in FGF21KO diabetic mice at the 2^nd^ month after diabetes onset. At the 4^th^ month after diabetes onset, FGF21KO diabetic mice showed aggravated aortic cell apoptosis when compared to WT diabetic mice (*P* = 0.079). However, FGF21 deficiency did not induce aortic cell apoptosis under non-diabetic conditions (Fig. 4a&b).

### FGF21 deletion exacerbated diabetes-induced aortic oxidative stress

**Fig. 5:**
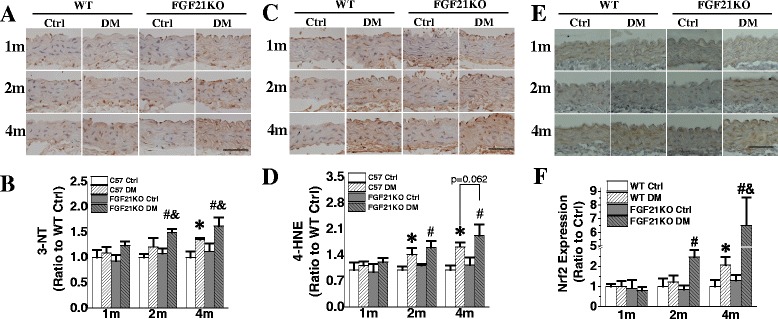
FGF21 deficiency accelerated and aggravated diabetes-induced oxidative stress. At indicated time points after diabetes onset, oxidative stress was evaluated by immunohistochemical staining of 3-NT (**a, b**) and 4-HNE (**c, d**). Antioxidative response was also evaluated by immunohistochemical staining of transcription factor Nrf2 (**e,f**). Data are presented as means ± SD, n ≥ 5 for each group. * *p* < 0.05 vs WT Ctrl group; # *p* < 0.05 vs FGF21KO Ctrl group; & *p* < 0.05 vs WT DM group. Bar = 50 μm. Abbreviations are the same as the Fig. 1

Excessive oxidative stress is considered a critical cause of aortic cell apoptosis and inflammation [25, 27]. Aortic oxidative stress was evaluated by measuring the accumulation of 3-NT and 4-HNE. FGF21KO mice did not show marked alterations in 3-NT accumulation under non-diabetic conditions (Fig. 5). Significant elevation of 3-NT accumulation was observed at the 4^th^ month in WT diabetic mice, and from the 2^nd^ month after diabetes onset in FGF21KO diabetic mice. Moreover, the aortic 3-NT accumulation in FGF21KO diabetic mice was significantly higher than that of WT diabetic mice at 2 and 4 months after diabetes onset (Fig. 5a&b). A similar pattern was observed in 4-HNE accumulation (Fig. 5c&d). The accumulation of 4-HNE in both WT and FGF21KO diabetic mice elevated since the 2^nd^ month after diabetes onset compared to the corresponding control mice. FGF21 deletion obviously exacerbated aortic 4-HNE accumulation at the 4^th^ month after diabetes onset compared to that of the WT diabetic mice (*P* = 0.062).

Nrf2, a transcription factor in regulation of various antioxidative and cytoprotective responses, has been shown to play an important role in cellular prevention against oxidative stress and damage *in vitro* and *in vivo* [25]. In FGF21KO diabetic mice, the aortic Nrf2 expression was significantly up-regulated, especially at the 4th month (Fig. 5e&f), indicating an adaptive response to the aggravated oxidative stress.

### FGF21 deletion exacerbated diabetes-induced impairment of eNOS activation

**Fig. 6:**
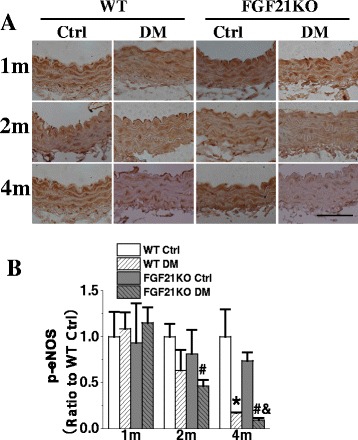
FGF21 deficiency accelerated and aggravated the impairment of eNOS activation in diabetes. At indicated time points after diabetes onset, eNOS activation was evaluated by immunohistochemical staining of p-eNOS (Ser-1177) (**a, b**). Data are presented as means ± SD, n ≥ 5 for each group. * *p* < 0.05 vs WT Ctrl group; # *p* < 0.05 vs FGF21KO Ctrl group; & *p* < 0.05 vs WT DM group. Bar = 50 μm. Abbreviations are the same as the Fig. 1

Nitric oxide synthase (NOS) is the pivotal enzyme in the production of nitric oxide (NO), which plays an essential role in vascular homeostasis as the elusive endothelium-derived relaxing factor [28]. eNOS is one of the major isoform of NOS existing in endothelium. eNOS-derived NO serves important functions including the regulation of vascular tone and regional blood flow, suppression of vascular smooth muscle cell proliferation, modulation of leukocyte endothelial interactions and thrombosis [29]. The activity of eNOS is promoted by phosphorylation at Ser-615, Ser-633 or Ser-1177, but inhibited by phosphorylation at Thr-495 [30]. Herein, we found that diabetes significantly inhibited aortic eNOS phosphorylation at Ser-1777 in WT diabetic mice at the 4^th^ month, and from the 2^nd^ month after diabetes onset in FGF21KO diabetic mice. Moreover, FGF21 deletion further attenuated the aortic eNOS function in FGF21KO diabetic mice compared to that of WT diabetic mice at 2 and 4 months after diabetes onset (Fig. 6).

### FGF21 administration ameliorated diabetes induced aorta dysfunction

**Fig. 7:**
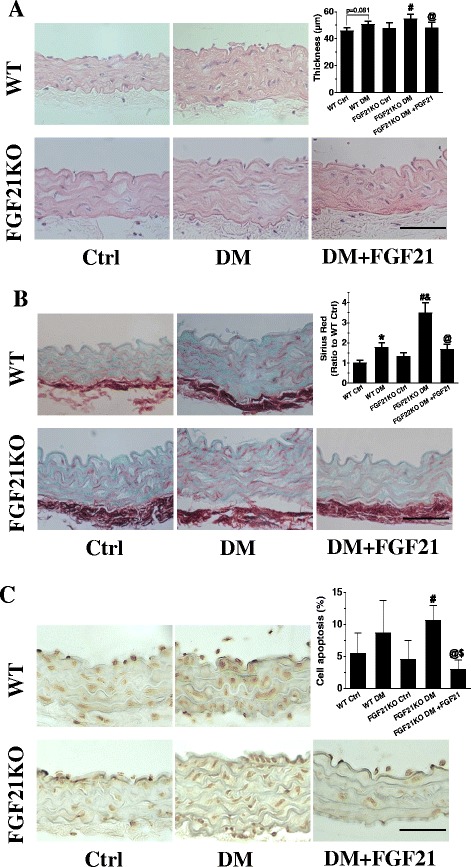
FGF21 administration reversed pathologic changes in the aorta of FGF21KO DM mice. In a type 1 diabetes model, FGF21 administration reversed aortic thickening (**a**), fibrosis (**b**) and cell apoptosis (**c**) in the aorta of FGF21KO DM mice. Data are presented as means ± SD, n ≥ 5 for each group. * *p* < 0.05 vs WT Ctrl group; #: *P* < 0.05 vs. FGF21KO Ctrl; @: *P* < 0.05 vs. FGF21KO DM; $: *P* < 0.05 vs. WT DM. Bar = 50 μm. Abbreviations are the same as the Fig. 1. FGF21KO DM + FGF21: FGF21KO DM mice received FGF21 administration

FGF21 deficiency aggravated aorta dysfunction induced by diabetes, and then we investigated whether FGF21 administration can reverse this process. In an acute type1 diabetes model, we found that FGF21 administration can ameliorate aortic thickening (Fig. 7a) and fibrosis (Fig. 7b) in FGF21KO diabetic mice. FGF21 treatment can also reverse cell apoptosis in FGF21KO diabetic mice. Moreover, cell apoptosis in the aorta of FGF21 treated FGF21KO diabetic mice was even lower than that in WT diabetic mice (Fig. 7c).

## Discussion

The therapeutic effect of FGF21 on diabetes and diabetic complications has been widely appreciated [13, 14]. But its effect on diabetic vasculopathy remains largely unknown. High level expression of the preferred receptor, FGFR1c, and co-receptor, β-klotho, in the aorta [22] indicates that aorta is a potential target tissue of FGF21. By using the FGF21KO mouse model, we provided the first experimental evidence to show that FGF21 deletion further accelerated and aggravated diabetes-induced aortic thickening, fibrotic remodeling, inflammation, cell apoptosis and oxidative stress, and FGF21 administration can reverse the pathologic changes in FGF21KO diabetic mice.

FGF21 has been considered as a potent regulator in glucose and lipid metabolism. The fact that blood glucose and lipid levels are comparable in WT and FGF21KO diabetic mice [31] and that FGF21 deletion further aggravates of the aortic thickening (Fig. 1), fibrosis (Fig. 2), inflammation (Fig. 3), cell apoptosis (Fig. 4) and oxidative stress (Fig. 5), suggests that the detrimental effect of FGF21 deletion on the aorta is most likely mediated by its direct action in aortic tissues rather than secondary actions such as manipulating systemic glucose and/or lipid metabolism.

The endothelium, which consists of a metabolically active monolayer of endothelial cells covering the entire luminal surface of blood vessels, plays a fundamental role in maintaining vascular homeostasis. Endothelial dysfunction was considered as a starting point for macroangiopathy and microangiopathy in both type1 and type 2 diabetes which would trigger the development of diabetic vasculopathy [4]. Cell apoptosis was assessed as an initial step of endothelial dysfunction [32]. In the present study, we found that FGF21 deletion accelerated and aggravated diabetes-induced aortic cell apoptosis (Fig. 4). These results are consistent with recent studies that FGF21 inhibits endothelial cell apoptosis induced by oxidized low density lipoprotein [33] or high glucose [34], enhances cell viability and decreases the apoptotic cell death in human umbilical vein endothelial cells (HUVECs) caused by H_2_O_2_ stress induction *in vitro*, while improves the condition of atherosclerotic rats *in vivo* [35, 36].

Chronic inflammation and oxidative stress play important roles in the development and progression of various chronic vascular pathological changes, including endothelial remodeling and apoptotic cell death under diabetic conditions [37]. It has been shown that FGF21 plays an important protective role against alcoholic fatty liver disease [38], drug-induced hepatotoxicity [39], atherosclerosis [35] and diabetic nephropathy [40] through its anti-oxidative stress and/ or anti-inflammatory actions. Herein we found that FGF21 deletion aggravated diabetes-induced oxidative stress, inflammation and fibrotic remodeling in aortas, reflected in the exacerbated accumulation of 3-NT and 4-HNE (Fig. 5 a-d), expression of TGF-β and TNF-α (Fig. 3), and accumulation of collagen and CTGF expression (Fig. 2), respectively. These results are consistent with a previous report that FGF21 deletion markedly aggravated acetaminophen overdose-induced liver damage, which was accompanied by increased oxidative stress and impaired antioxidant capacities. The replenishment of recombinant FGF21 largely reversed acetaminophen-induced hepatic oxidative stress and liver injury in FGF21KO mice [39], and are concurrent with our previous studies that demonstrated FGF21 administration attenuates diabetes-induced oxidative stress and inflammation in testis [27] and kidney [41].

One possible cause for the aggravated pathological changes of the aorta in FGF21KO diabetic mice is the dysfunction of eNOS. eNOS gene deficiency resulted in hypertension [42], increased vascular smooth muscle cell proliferation in response to vessel injury [43], increased leukocyte-endothelial interactions [44], hypercoagulability [45] and increased diet-induced atherosclerosis [46]. Recently, an *in vitro* study [34] showed that eNOS phosphorylation at Ser-1177 and Ser-633 in HUVECs was impaired under diabetic conditions, which can be rescued by FGF21 administration in an AMP-activated protein kinase-dependent manner. In present study, phosphorylation of eNOS at Ser-1177 was further down-regulated in FGF21KO diabetic mice than that in WT diabetic mice (Fig. 6), which indicated that FGF21 deficiency may contribute to the aggravated aortic damage by impairing eNOS activation.

In conclusion, we found that FGF21 deletion accelerates and aggravates diabetes-induced aortic pathological changes reflected by exacerbated aortic thickening, collagen accumulation and fibrotic remodeling, which is most likely due to FGF21 deficiency-induced aggravation of aortic oxidative stress, inflammation, and cell apoptosis, and FGF21 administration can reverse those pathologic changes in FGF21KO diabetic mice.
